# Sex Differences in Aortic Stenosis: From the Pathophysiology to the Intervention, Current Challenges, and Future Perspectives

**DOI:** 10.3390/jcm13144237

**Published:** 2024-07-19

**Authors:** Paolo Springhetti, Kathia Abdoun, Marie-Annick Clavel

**Affiliations:** 1 Institut Universitaire de Cardiologie et de Pneumologie de Québec, Université Laval, Québec, QC G1V 4G5, Canada; paolo.springhetti.1@ulaval.ca (P.S.); kathia.abdoun.1@ulaval.ca (K.A.); 2 Department of Medicine, Division of Cardiology, University of Verona, 37129 Verona, Italy

**Keywords:** aortic stenosis, sex differences, diagnosis, complications, outcomes, aortic valve replacement, patient–prosthesis mismatch

## Abstract

Calcific aortic stenosis (AS) is a major cause of morbidity and mortality in high-income countries. AS presents sex-specific features impacting pathophysiology, outcomes, and management strategies. In women, AS often manifests with a high valvular fibrotic burden, small valvular annuli, concentric left ventricular (LV) remodeling/hypertrophy, and, frequently, supernormal LV ejection fraction coupled with diastolic dysfunction. Paradoxical low-flow low-gradient AS epitomizes these traits, posing significant challenges post-aortic valve replacement due to limited positive remodeling and significant risk of patient–prosthesis mismatch. Conversely, men present more commonly with LV dilatation and dysfunction, indicating the phenotype of classical low-flow low-gradient AS, i.e., with decreased LV ejection fraction. However, these distinctions have not been fully incorporated into guidelines for AS management. The only treatment for AS is aortic valve replacement; women are frequently referred late, leading to increased heart damage caused by AS. Therefore, it is important to reassess surgical planning and timing to minimize irreversible cardiac damage in women. The integrity and the consideration of sex differences in the management of AS is critical. Further research, including sufficient representation of women, is needed to investigate these differences and to develop individualized, sex-specific management strategies.

## 1. Introduction

Calcific aortic valve stenosis (AS) is the most common valvular heart disease requiring intervention in high-income countries [1] and the AS global burden is expected to grow in the next years due to the progressive aging of the population [2]. As medical research progresses, it is becoming increasingly clear that men and women do not share the same anatomic features, but they also show differences in the manifestation and progression of AS. These disparities raise important questions about diagnostic approaches, management, and sex-specific clinical management. Both the actual European [3] and American Guidelines [4] report class I indication for aortic valve replacement (AVR) for severe symptomatic AS or severe asymptomatic AS, if this latter is accompanied by left-ventricular (LV) dysfunction or coexists with an indication for other concomitant cardiac surgery. In asymptomatic severe AS there is a growing body of evidence regarding risk stratification through additional markers such as pro N-Type Brain Natriuretic Peptide dosing [5], the detection of very severe AS (peak aortic jet velocity (V_peak_) > 5 m/s or mean gradient > 60 mmHg) [6], and exercise stress testing [7], aiming to identify patients who could potentially benefit from early AVR (class II indications). In the context of asymptomatic AS, speckle tracking echocardiography with both LV [8,9] and left-atrial strain assessment have been revealed as risk stratification tools [10]. The research focus now extends to optimal management in both severe and non-severe AS, exploring evidence for early AVR in other specific scenarios [11]. A recent study from Généreux P. et al. highlights that the risk of mortality increases continuously with the AS severity degree, starting from mild and moderate forms [12], according to the findings of Strange et al. [13], suggesting the currently unmet optimized management of non-severe AS. The trial PROGRESS (NCT04889872) is ongoing, aiming to specifically evaluate the safety and effectiveness of transcatheter aortic valve replacement (TAVR) also in moderate AS. The consideration of pseudo-severe AS, a specific phenotype characterized by low flow, LV dysfunction and severely reduced aortic valvular area despite non-severe confirmation at second-line imaging, is important. While not a primary indication for AVR, a recent multicentric retrospective study by Ludwig S. et al. revealed that TAVR is a significant predictor of improved long-term outcomes among patients with pseudo-severe AS [14]. This emphasizes the necessity for further research also in this particular setting. All these described scenarios are focusing on researching the best early aortic intervention timing, but this aspect is facing some other big challenges. Recent findings underscore that women with severe symptomatic AS are often referred later for aortic valve intervention, leading to increased mortality and cardiovascular events [15]. This unfavorable condition is amplified by the evidence that more advanced extracardiac valvular damage [16], higher surgical risk scores [17], and concomitant low-flow statuses are observed in women undergoing AVR [18], so future research is warranted to equally optimize AS management in both sexes. The aim of this review is to focus specifically on the female-related AS context from a pathophysiological and clinical point of view, also encompassing the management of AS and considerations of possible future perspectives.

## 2. Aortic Valve Anatomy

**Figure 1:**
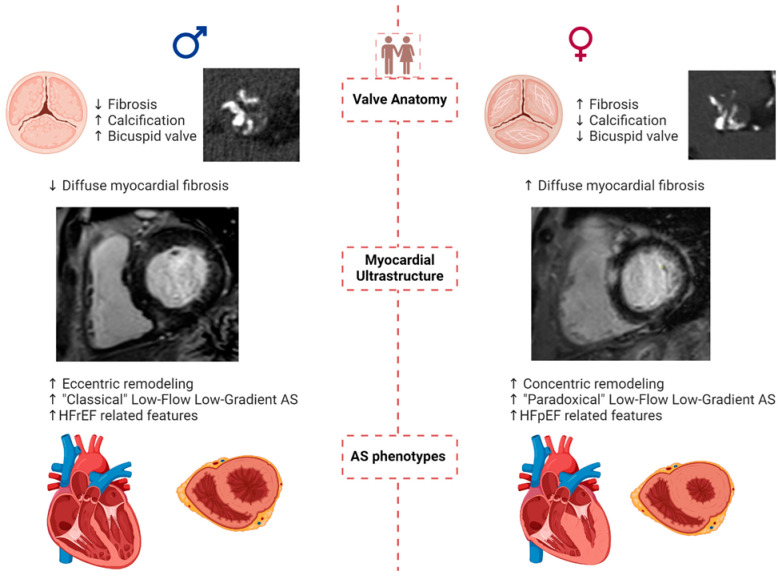
Pathophysiology and presentation of aortic stenosis in women and men. Legend: AS: aortic stenosis; HFrEF: Heart Failure with reduced Ejection Fraction; HFpEF: Heart Failure with preserved Ejection Fraction.

Women present a smaller aortic annulus than men and a shorter distance between the annulus and the coronary ostia, corresponding in part to their smaller body surface area [19]. As a matter of fact, even with similar body surface area, women have smaller hearts [20]. The incidence of AS is higher in males than in females for younger patients due to a higher prevalence of congenital bicuspid aortic valve (BAV), which has a male-to-female ratio of 3:1 (Figure 1) [21,22,23]. Based on systematic echocardiographic screening, the prevalence of BAV is estimated to be 0.6% to 0.8% in men and 0.2% in women [24,25]. Currently, no difference has been found in the distribution of phenotypic types of bicuspid valve morphology between the two sexes [26], but data remain insufficient regarding this topic. 

After the age of 75 years, the incidence of AS is reversed and slightly higher in women [27].

## 3. Calcification and Fibrosis of the Aortic Valve According to Sex

Valvular lesions in AS were, for a long time, considered to be similar between sexes, and male sex was identified as a predictive factor for occurrence and hemodynamic progression of AS. However, previous research has underrepresented the female sex, leading to a lack of conclusive results [21]. Several recent studies have examined the sex-related AS differences, including hemodynamic severity and aortic valve calcification (AVC), and valve remodeling.

AVC, which is the primary lesion of calcific AS, could be assessed by computed tomography (CT) and correlates with hemodynamic severity measured by echocardiography. AVC is a powerful determinant of the severity of AS and a major risk factor for AS progression and adverse outcomes [28,29,30]. For a similar hemodynamic AS severity, women have less aortic valvular calcification and higher levels of valvular fibrosis (Figure 1) with more dense connective tissue [31,32,33,34,35]. Consequently, specific calcification score thresholds of 1200 or 1300 Agatston units for women and 2000 Agatston units for men have been established to identify severe AS [3,4,28,36]. 

The impact of sex on the mechanism of AVC remains poorly understood. In the pathophysiology of AVC, inflammation, lipoprotein profile, and matrix remodeling are the main factors involved in the calcification process [37]. Additionally, gene expression and hormonal status, particularly testosterone, has been implicated in vascular smooth muscle culture calcification and AS progression in animal models [38,39,40,41,42,43]. These differences could potentially explain the variation and underlying mechanisms that may differentiate AVC burden between sexes. Mechanisms underlying sex differences in fibrosis include different gene expression profiles and phenotypes [41]. Valvular fibrosis in women may be attributed to enhanced activation of the myofibroblasts pathway in the interstitial cells of the aortic valve associated with genes that escape X-chromosome inactivation [44]. 

Due to various limitations and underrepresentation of women in studies, it remains challenging to draw definitive conclusions regarding potential differences in hemodynamic or anatomic progression of AVC between men and women. In the Simvastatin Ezetimibe in Aortic Stenosis (SEAS) study, the annual hemodynamic progression of mild to moderate AS was found to be similar between men and women [45]. Similar results were observed by Tastet et al. in a study with a similar patient profile; however, the correlation slope between mean gradient progression and AVC was more pronounced in women than in men [30]. The COFRASA-GENERAC study demonstrated that female sex was an independent predictor of both hemodynamic and anatomic progression [46]. These results underscore a distinct progression profile between men and women in AS. However, increased female participation is necessary to deepen our understanding of the impact of sex on AS progression. This will also help to guide clinical practice in terms of follow-up timing and diagnosis.

## 4. LV Remodeling, Comorbidities, and AS Presentation According to Sex 

AS has, for a long time, been described as a pathological condition which involves both the valve and the LV as a consequence of remodeling against chronic increased afterload [47,48]. Geometric patterns of LV include a normal pattern, concentric remodeling, concentric hypertrophy, and eccentric hypertrophy [49,50]. LV concentric remodeling with concomitant smaller cardiac chambers and lower LV mass is more prevalent in women [19,51]. Concentric hypertrophy was associated with worse clinical outcomes in women (Figure 1) [51]. Men have a higher prevalence of both concentric and eccentric hypertrophy across the spectrum of AS severity [52,53]. Some pivotal echocardiographic studies have shown that women present greater LV thickness than men both in severe [54,55] and non-severe [47,56] AS. In addition, women develop a restrictive LV pattern and are more likely than men to develop heart failure in response to cardiac overload [57]. 

CMR findings on myocardial fibrosis and sex differences are discordant. Dobson LE et al. found no sex difference in late gadolinium enhancement (LGE), indicating long-standing fibrosis [58], whereas Treibel TA et al. observed more LGE in men [53]. More recently, Tastet et al. [16] reported, in patients with a similar amount of LGE between sexes, a higher extracellular volume in women, suggesting diffuse fibrosis [59,60]. The discordance in findings may be attributed to differences in patient’s baseline characteristics across studies. Tastet et al.’s cohort [16] included a wider range of AS severity, different from other research that has been mainly focused on severe symptomatic AS. Further sub-analyses of this study provided compelling insights regarding the development of fibrosis, demonstrating that women exhibit fibrosis from earlier AS stages, a process driven by the renin–angiotensin–aldosterone system [61,62]. LV fibrosis and concentric remodeling are prominent features of paradoxical low-flow (PLF) AS, characterized by reduced cardiac output despite preserved ejection fraction. Due to the low-flow state, low-gradient (<40 mmHg) and small AVA (<1 cm^2^) are often concomitant [63]. This phenotype is highly prevalent in hypertensive women (Figure 1) and may be a reason for underdiagnosis and underestimation of AS severity in women [18,64]. PLF is associated with negative outcomes [64,65,66]. On the other hand, men are more frequently affected by coronary artery disease and reduced LV ejection faction (LVEF), i.e., classical low flow (CLF), which is also associated with low-gradient/small AVA, and worse outcomes [21]. The prevalence of PLF AS (up to 20%) is higher than the prevalence of CLF (up to 10%) AS [67]. 

Scarsini R et al. found that patients with PLF low-gradient AS often exhibit severe microcirculatory dysfunction and reduced peak atrial longitudinal strain [68], with the latter being a potential indicator of LV fibrosis [69] and yielding significant prognostic information [70] in AS. Guzzetti et al. also recently described a PLF high-gradient AS phenotype, associated with a worse prognosis than PLF low-gradient AS and related to sex-specific stroke volume index thresholds linked to a better risk stratification [71]. Further mechanisms combined with the increased afterload imposed by AS have been described as responsible for the concentric remodeling tendency observed in women.

Hypertension could play an important role [15] not only in sustaining concentric LV remodeling [72] and diastolic heart failure with preserved ejection fraction [73] but also yielding challenges in echocardiographic evaluation, potentially leading to a normal-flow low gradient, despite reduced aortic valve area presentation, which can be misleading and result in underestimation of AS [74]. There is concordance in the literature regarding the increased prevalence of diastolic dysfunction in women with AS in comparison to men [16,75], regardless of AS severity [76]. 

Dayan et al. [77] identified that PLF low-gradient AS shares similar features with Heart Failure with preserved Ejection Fraction (HFpEF), such as diastolic dysfunction and concentric remodeling, with altered ventricular–arterial coupling being a crucial link between the two conditions [78,79]. The H_2_FPEF score, validated for identifying likely HFpEF [80] also correlates with increased prevalence of PLF AS [81] and is associated with poorer exercise capacity and adverse hemodynamics in moderate to severe AS [82]. 

However, a recent position paper categorizes valvular heart disease as a “HFpEF mimic” rather than true HFpEF, naming it “HF attributed to valvular heart disease” to distinguish it from HFpEF [83]. De Biase N et al. recently identified overlaps in demographic–clinical characteristics, functional capacity impairment, and epicardial adipose tissue accumulation patterns between PLF AS and HFpEF [84]. These aspects, as reported by the authors in the conclusion [84], reinforce the hypothesized concept of PLF AS as a specific HFpEF sub-phenotype and warrant further investigation.

Conversely from the more widespread tendency of concentric remodeling, diastolic impairment, and PLF AS evolution observed in women, the progression towards LV dilation and heart failure with reduced ejection fraction (HFrEF) are features linked mainly to long-standing AS in men [85]. Men represent approximately 70–75% of patients with CLF AS [86,87], regardless of the severity degree. Coronary artery diseases, more commonly observed in men, could concurrently enhance the evolution towards LV dilatation and dysfunction in AS [88].

## 5. Multimaging in AS Evaluation

Clinical examination and non-invasive imaging assessment are essential in the diagnostic process of AS, not only focusing on valve structure and function but also enabling extra-valvular cardiac damage detection, facilitating the decision-making process in routine clinical practice. 

### 5.1. Transthoracic Echocardiography (TTE)

**Figure 2:**
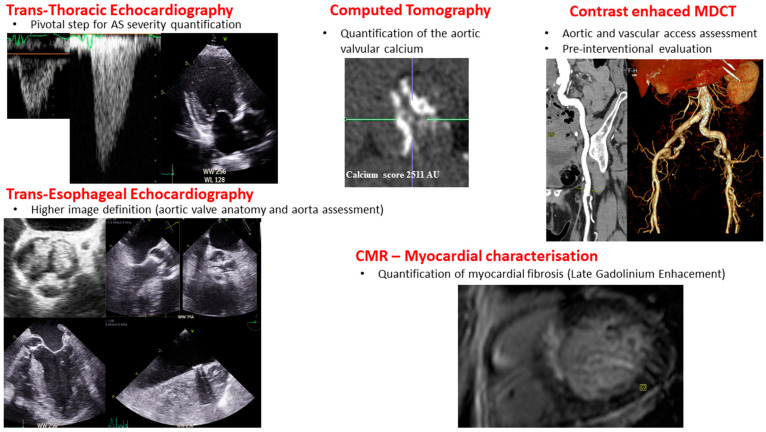
Multi-imaging modalities in aortic stenosis diagnosis. Legend: AS: aortic stenosis, CMR: Cardiac Magnetic Resonance, MDCT: Multi-Detector Computed Tomography.

Two-dimensional TTE is the primary imaging modality for the assessment, diagnosis, and severity grading of AS, and it enables evaluation of AV morphology—as shown in Figure 2 [89,90,91,92,93]. V_peak_, mean transvalvular gradient, and AVA are the main echocardiographic parameters used to determine the severity of AS. AVA can be indexed to account for size differences, especially in small patients. Other parameters could be assessed in case of uncertainty or for risk stratification, such as the Doppler velocity index, valvulo-arterial impedance, stroke volume index, etc. TTE is also used to assess both LV structure and function. Calculation of LV mass from ventricular diameter measurements is used to assess the hypertrophic response in AS [89]. 

Women typically have smaller LV dimensions than men, resulting in a smaller LV mass [94,95,96,97,98,99]. Global longitudinal strain (GLS) is an important parameter in the evaluation of LV contractile function. Several studies have demonstrated the prognostic value of this imaging biomarker in predicting cardiovascular events, morbidity, and mortality in patients with AS, especially when the LVEF is preserved [89,100,101,102,103,104,105]. Heterogeneity in GLS cut-offs is present in the literature, and it is also partially dependent on the software utilized for speckle-tracking analysis. Nevertheless, a GLS threshold between −18% and −16% finds agreement by most studies [106,107,108]. The adoption of GLS could be useful in clinical practice for the risk stratification of patients with asymptomatic severe AS with preserved LV ejection fraction, not strictly presenting a Class I indication for AVR [8,106]. 

GLS and amyloidosis identification in the setting of AS is controversial since the presence of the so-called “apical sparing” pattern is also present in advanced remodeled LV [109]. A multi-imaging approach associated with careful clinical evaluation is key to resolve this issue [110]. Indeed, amyloidosis often presents often with low-gradient, small AVA and lower aortic valve calcium burden than expected, as well as granular myocardial texture, severe diastolic dysfunction, and specific clinical manifestations (e.g., renal disease, neuropathy, disturbance of cardiac conduction).

Although TTE remains the primary tool for diagnosing AS and monitoring its progression, it has certain limitations. Other advanced imaging modalities play an important complementary role, allowing us to examine a patient’s cardiac health with enhanced precision (Table 1 and Table 2).

### 5.2. Dobutamine Stress Echocardiography (DSE)

DSE as a second-line imaging method is indicated in patients with low-flow, low-gradient AS with depressed LV ejection fraction to establish the AS severity. Reduced LVEF with AS is more common in men. Although DSE is safe in patients with preserved ejection fraction, its accuracy in patients with mildly reduced or normal LVEF is lower than in patients with severely reduced LVEF [111]. Moreover, the use of mean gradient > 40 mmHg and AVA < 1 cm^2^ at any stage of DSE to confirm severe AS has been shown to be inconclusive or inaccurate in many patients, as many patients do not reach normal flow during DSE [112]. Hence, the measurement of aortic valve calcium score (AVC) should be preferred, especially in case of PLF LG. 

### 5.3. Computed Tomography (CT)

CT is a valuable imaging modality for anatomical assessment, allowing precise measurement of AVC that is a truly flow-independent marker of AS severity—as shown in Figure 2 [36,90,113]. AVC score and volume are measured on a non-contrast ECG gated CT scan [36,90,114]. Calcium scoring measurements are highly reproducible markers of LV decompensation that demonstrate excellent agreement with echocardiographic measurements and serve as a robust predictor of future clinical events, outperforming echocardiography in all patient groups, even those with discordant classification, CLF, and/or PLF [29,90,114,115,116]. Sex-specific thresholds (i.e., 1200 or 1300 Agatston units for women and 2000 Agatston units for men) have been validated in several international cohorts and are used to predict AS progression and adverse clinical events [36,90,117]. In order to take into account aortic valve size, AVC could be indexed to the cross-sectional area of the aortic annulus, i.e., the AVC density, which is mostly useful in patients with small or large annulus, such as patients with bicuspid valve [28,36,118].

Contrast-enhanced CT can enhance the anatomical evaluation of AS severity and provides several advantages compared to non-contrast examination [90,119]. In a post hoc analysis by Cartlidge et al., contrast-enhanced CT assessment of calcified and non-calcified volumes in the aortic valve showed correlations with the severity of AS [119]. Further studies are needed to develop a reliable methodology and establish severity thresholds to assist in clinical decision-making.

Finally, contrast-enhanced CT is also the cornerstone of the peripheral vascular evaluation pre-procedural strategy (Figure 2), which is especially useful in women where iliofemoral dissections and perforations are more common than men [120], as sex differences also exist in terms of the access difficulty for a transcatheter approach. 

### 5.4. Positron Emission Tomography (PET Scan)

Although CT can identify anatomical lesions in AS, it does not provide information about the calcification process. PET scans can be used to measure the activity of the calcification process [121]. Radiotracers are injected intravenously and localize in areas where the pathological process of interest is active. Valvular calcification activity in AS is evaluated using 18F-fluoride [90]. Dweck et al. demonstrated that 91% of patients with AS had increased 18F-NaF uptake, with a progressive rise in tracer activity correlating with AS severity [122]. In addition, longitudinal studies have shown that baseline 18 F-NaF uptake is a predictor of AS progression and AVR [123,124]. The use of 18F-NaF PET may be beneficial for bioprosthesis evaluation and early detection of degeneration. In observational studies, 18F-NaF uptake has been demonstrated as an independent and early predictor of bioprosthesis degeneration, outperforming other factors such as echocardiographic findings [125,126]. While PET scans enable early detection of AVC, their clinical utility in AS is limited. Indeed, CT provides similar prognostic information at a reduced cost and with lower radiation exposure [90].

### 5.5. Cardiac Magnetic Resonance (CMR)

CMR is currently adopted to evaluate myocardial-related features (Figure 2), particularly through late gadolinium enhancement and extracellular volume mapping assessment, providing structural information and myocardial tissue characterization, as discussed in the LV remodeling paragraph [90].

## 6. Symptoms and Clinical Profile in AS: Sex Differences 

Women are more likely to present nuanced symptoms compared to men, often complaining of less specific symptoms such as shortness of breath and dizziness. This trend may be explained by a higher prevalence of microvascular dysfunction, a higher frequency of concomitant tricuspid/mitral valve disease, a smaller LV cavity, and a lower LV mass associated with diastolic dysfunction. Additionally, women have a shorter duration of exercise and a lower anaerobic threshold [15,97,127,128]. On the other hand, men are more likely to suffer from angina, which may be due to the higher incidence of coronary heart disease in men [19]. In studies of severe AS, while men had atherosclerotic comorbidities, particularly coronary artery disease, women were older, more often frail, had a higher Society of Thoracic Surgeon (STS) score, and a higher prevalence of hypertension and chronic obstructive pulmonary disease [19]. Moreover, women are more likely than men to develop symptoms during follow-up despite similar initial severity of AS [97]. 

Finally, the delay to diagnosis and treatment in women could also be associated with gender bias, as symptoms and AS severity in women are often undermined by physicians. Indeed, delay to intervention has been shown in women with similar AS severity and symptoms than men [15]. However, sex-and gender-specific studies in AS are needed to confirm this point. 

## 7. AVR Referral: Surgical AVR, TAVR, and Ross Procedure

### 7.1. Surgical AVR (SAVR)

The onset of symptoms, which indicates LV decompensation and is associated with a poor prognosis in severe AS, is a trigger for AVR according to the guidelines [3,4]. Differences between sexes in the timing and manifestation of symptoms can significantly influence therapeutic decisions, long-term health preservation, and the risk of irreversible cardiac damage associated with delayed referral for AVR, peri/postoperative complications, mortality, and long-term outcomes. Consequently, it is crucial not to base therapeutic decisions solely on symptoms. Women are referred for AVR later than men, despite having more symptoms. Male patients are more likely to have Class I indications for AVR based on echocardiographic parameters [129,130]. The tendency for men to be referred earlier for AVR is in part related to a higher frequency of concomitant procedures such as coronary artery bypass grafting or aorta repair/replacement. Discordance of echocardiographic parameters is a second explanation for delayed referral of female patients for AVR [15]. Another important reason for this lack of intervention is the conviction that symptoms are unrelated to AS, but unoperated patients are at higher risk of mortality [131]. 

Women exhibit higher rates of in-hospital mortality compared to men [132]. In the Society of Thoracic Surgeons’ national database, focusing on patients undergoing isolated SAVR, female patients had an increased risk of mortality, stroke, and postoperative hospitalization compared to male patients [133]. Another study showed that overall survival was worse in women than in men; however, after adjusting for preoperative risk factors, there was no significant difference in overall survival between the two sexes [134]. Fuchs et al. presented contrasting results, demonstrating that operative and long-term mortality did not increase in women after SAVR. Furthermore, women experienced better outcomes [135]. This discrepancy in outcomes may be attributed to pre-procedural comorbidities and cardiac damage, as well as the PLF AS [15,51,71,132]. 

### 7.2. TAVR 

**Figure 3:**
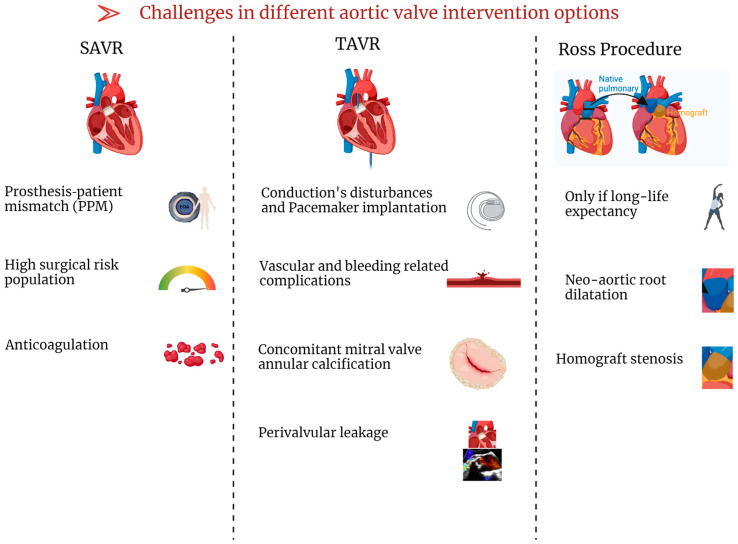
Challenges in the different aortic valve intervention options. Legend: SAVR: surgical aortic valve replacement; TAVR: transcatheter aortic valve replacement.

Women are more likely to undergo TAVR [17,19]. Overall, women generally have fewer cardiovascular risk factors and less calcium burden, with lower rates of prior myocardial infarction, revascularization, prior stroke, and peripheral vascular disease. Additionally, they often have better LVEF at presentation [19,21]. Several studies suggest that TAVR offers significant benefits to women. In the CoreValve US High-Risk Pivotal Trial, women treated with TAVR had lower one-year all-cause mortality compared to those undergoing SAVR (12.7% vs. 21.8%) [136]. In the majority of TAVR studies, women had comparable or favorable results to men after one or two years [137,138,139,140,141,142,143,144,145,146,147,148]. Women have a lower risk of developing paravalvular regurgitation, an important prognostic factor after TAVR [149,150], than men [151,152]. Less AVC and a smaller annular size in women may explain this difference in results [153]. In contrast to men, women had a higher incidence of major bleeding and in-hospital vascular complications after TAVR (Figure 3), as well as device-related complications, stroke, and conversion to conventional SAVR [133,140,141,154,155], but with a lower rate of re-hospitalization [156]. The increased incidence of vascular complications and major bleeding in women undergoing TAVR may be due to their advanced age and the relatively smaller dimensions of the vessels, annulus, and LV ejection pathway [19,157]. The most recent study regarding the non-inferiority of TAVR in low-risk, symptomatic severe AS—the PARTNER trial—presents results after 5 years of follow up [158]. There is a tendency of more adverse outcomes after SAVR in women shown by different studies [159,160,161,162], potentially representing pivotal clinical issues in favor of TAVR in women with severe AS requiring intervention. Furthermore, a specific multicenter register (Women’s International Transcatheter Aortic Valve Implantation [WIN-TAVR] Registry) including only woman with intermediate to high risk undergoing TAVR [163] reported a 1-year VARC-2 efficacy endpoint of 16.5% with low incidence of 1-year mortality and stroke rate of 13.9%. A subsequent recent sub-analysis highlights that the VARC-2 endpoint was higher and mostly sustained by patients with concurrent frailty/prefrailty criteria [164]. Nevertheless, there is not yet available evidence from randomized studies regarding the superiority of TAVR compared to SAVR in women with symptomatic AS, independently from surgical risk. An ongoing randomized multicentric controlled trial has been specifically designed in order to clarify this aspect (Randomized researcH in womEn all comers wIth Aortic stenosis [RHEIA] trial) [165].

### 7.3. Ross Procedure

The Ross procedure appears to be beneficial and particularly appropriate for young women [166], especially those considering pregnancy [167]. This is mainly due to (1) the advantage of avoiding the need for anticoagulation, which can be a challenge in managing pregnancy for both maternal and fetal health [168] and (2) the longer valve durability of the autograft (from Ross procedure) compared to bioprostheses [169]. Indeed, several studies have reported various cardiovascular and obstetric complications in pregnant women with mechanical valve replacement, and thus bioprostheses are mainly implanted in young women planning pregnancy. However, studies have shown no adverse associations between pregnancy and the Ross procedure in pregnant women [170,171,172,173]. It is important to highlight that, despite the generally favorable tolerance of pregnancy in these women, there may be a risk of neo-aortic dilatation (Figure 3), especially in those who have had multiple pregnancies [173,174]. As women have unique characteristics, a special approach is required, particularly with regard to pregnancy planning given the potential risks to maternal and fetal health. Individual counseling prior to conception in young women with bicuspid aortic valve is essential to assess risks and discuss options. Close follow-up during pregnancy is essential to evaluate disease progression and make any necessary treatment adjustments while minimizing risks to both the mother and the fetus. In addition, postpartum and long-term follow-up are required to assess and prevent long-term complications. 

Specific indications for the three types of interventional treatment for AS are shown in Table 3.

**Figure 4:**
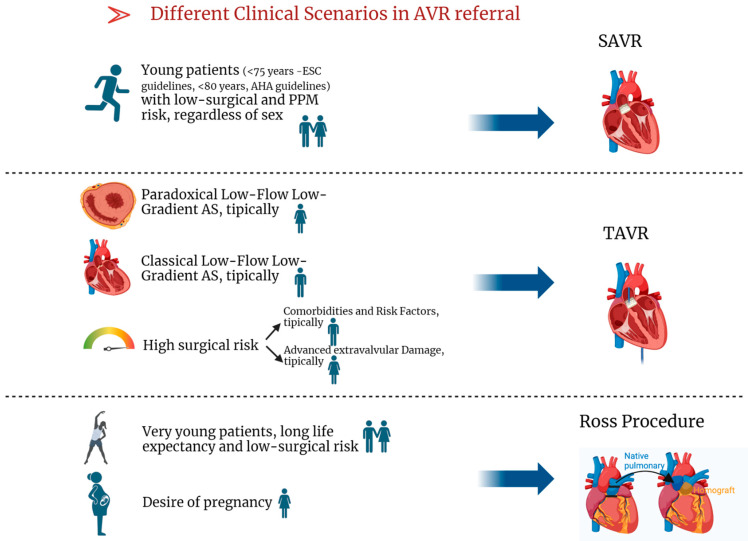
Different Clinical Scenarios in AVR referral. Legend: AS: aortic stenosis, AVR: aortic valve replacement, SAVR: surgical aortic valve replacement, TAVR: transcatheter aortic valve replacement.

Figure 4 aims to summarize common scenarios of severe AS encountered in clinical practice referred for AVR and their proposed best treatment option. 

## 8. Current Challenges in AVR

The planning of aortic valve intervention has some important aspects: Prosthesis–patient mismatch (PPM) occurrence is more common after SAVR, while conduction disturbance and vascular/bleeding complications are mostly observed in TAVR. Also, the presence of severe mitral annular calcification represents an important field of research because evidence in the literature regarding its prognostic role after TAVR is discordant.

### 8.1. Prosthesis–Patient Mismatch (PPM)

PPM after AVR occurs when the effective orifice area (EOA) is too small for the patient’s cardiovascular requirements [175,176]. Severe PPM after SAVR occurs in 11% of patients, mostly in women (57%) [177,178,179,180]. Recently, the definition of PPM has been updated, lowering the threshold for patients with a body mass index (BMI) above 30 kg/m^2^ (VARC 3 Definition) [181,182], which decreases the prevalence of severe PPM to 3% [183]. Severe PPM is less common after TAVR than SAVR, regardless of the definition [184,185,186].

In clinical practice, a small aortic annulus (less than 400 mm^2^) in patients with severe AS represents a significant challenge [187]. This condition is identified as a risk factor for PPM following AVR [188,189], and it predominantly affects elderly women with a small LV cavity and concentric remodeling [187,190]. Choosing SAVR over TAVR in such cases may potentially increase the risk of PPM (Figure 3), leading to poorer long-term clinical outcomes [191]. The VIVA trial, enrolling 151 patients, showed no difference in medium-term outcomes between SAVR and TAVR in the context of severe AS with small annulus [192]. However, in women with PLF AS, TAVR may improve outcomes compared to SAVR [193]. The RHEIA multicentric randomized trial aims to further elucidate specifically whether TAVR is a better option than SAVR in women with small annulus, regardless of the patients’ baseline clinical surgical risk [165]. The minimization risk of PPM after AVR is important especially in patients with CLF [194,195] or PLF AS [196], which are specific phenotypes where PPM contributes significantly to worse outcomes [196,197]. 

Severe PPM should be avoided in all patients and in certain “vulnerable subsets” even moderate PPM should be avoided, such as those with CLF AS, PLF AS, severe LV hypertrophy, and mitral regurgitation [191]. The occurrence of significant PPM could be predicted before (or during) the procedure, using published EOA values for the type and size of the prosthesis that is planned to be implanted according to the aortic annulus size measured by CT or echocardiography [198] and indexed to the body surface area. For “vulnerable patients” at risk of severe or moderate PPM, options to avoid PPM include selecting an alternative prosthesis (with better hemodynamics), aortic root enlargement, or consideration of TAVR, which presents lower PPM prevalence [191]. 

### 8.2. Pacemaker Implantation 

While permanent pacemaker implantation was common in women after TAVR in the WIN-TAVR registry [199], a metanalysis involving 46 studies and encompassing more than 70,000 patients with TAVR highlighted that pacemaker implantation incidences occur less frequently in women, suggesting that conduction disturbances such as TAVR-related complications are more common in men [200]. This issue could be partially explained by the lower calcium burden detected in women with AS, especially in the non-coronary cusp [201]. The anatomical proximity of both the atrium-ventricular node and left Tawara branch to the non-coronary cusps, susceptible to compression during valve implantation, may explain this complication after TAVR. The aortic calcium burden is additionally linked to the development of paravalvular leak after TAVR. Women are more likely to present paravalvular aortic leak than men, a feature linked to adverse long-term outcomes [151,152,153].

Finally, other important anatomic determinants of pacemaker implantation after TAVR are very severe reduced aortic valve area (below 0.75 cm^2^) [202], differences between implantation depth and membranous septum length above 3 mm [203], greater aortic annulus diameter, and the presence of mitral annular calcification [204].

### 8.3. Frailty and Bleeding Risk 

A particular phenotype of AS where TAVR in women demonstrates poorer outcomes than in men is represented by low-flow, low-gradient AS [205]. This trend was also observed in patients who were very elderly and frail [206], as well as in women reporting coexistent pulmonary hypertension [207]. Additionally, some studies report that women are often a technically challenging subset of patients for TAVR, also exhibiting more vascular complications and bleeding in the post-operative period (Figure 3) [208,209]. 

### 8.4. Mitral Annular Calcification 

The presence of severe mitral annular calcification (MAC) has been previously reported to be an independent predictor of long-term negative outcomes after TAVR. Severe MAC is mostly observed in women, and after adjustment for other clinical parameters it was associated with increased all-cause cardiovascular mortality and conduction disturbance after TAVR [210]. However, if MAC is an independent predictor of prognosis regardless of concomitant mitral valve disease (regurgitation or stenosis), its impact remains a matter of debate since the available evidence reports discordant findings [211,212,213].

## 9. Conclusions and Future Perspectives

Sex-related differences are encountered in AS, ranging from pathophysiology, encompassing extra-valvular damage, and the diagnostic process, up to intervention when needed [21,209]. The conclusions of this review can be summarized as follows:

(1) The calcific burden in AS is less represented in women, with more concomitant widespread fibrotic patterns sustained by different biological pathways.
(2) HFpEF and PLF AS, especially in woman, present common features, warranting further studies aiming to elucidate if PLF AS could be included in the HFpEF spectrum.
(3) Referrals for aortic valve intervention tend to be later in women, leading to an augmented extra-valvular damage-related burden.
(4) SAVR carries a higher risk of severe PPM compared to TAVR, especially in women, PLF AS, and concomitant small aortic anulus. TAVR in those scenarios could be preferred; ongoing studies are focusing on this strategy in women with small annulus.
(5) TAVR is facing some challenges in women, especially considering the bleeding risk, frailty and higher prevalence of mitral annular calcification.
(6) The Ross procedure performed in highly specialized centers is a valid option in patient with low surgical risk and without underlying comorbidities.

Further studies are required to deepen our knowledge of sex-specific AVC and structural heart changes, along with the creation of tailored antithrombotic treatments designed to lower the heightened risk of bleeding in women after TAVR [148]. Moreover, to enhance patient outcomes in AS, it is essential to establish sex-specific thresholds for early and accurate diagnosis in women [209]. It is also important to examine disparities in patient referrals to guarantee equal opportunities for both sexes with severe AS to access the best possible care [214]. Through these measures, we can devise individualized treatment plans for patients with severe AS accounting for sex differences, leading to better health outcomes.

**Table 1:** Advantages and limitations of the established imaging modalities used to assess AS.

	Indication/Diagnostic	Advantages	Limits
TTE [18,67,89,90,91,92,93,215,216,217,218,219]	– Diagnosis of AS and grading severity – Hemodynamic assessment – Analysis of flow dynamics, direction, and type (e.g., regurgitation) – Evaluation of LV structure and function – Assesses the structure and function of the other heart chambers and detects concomitant abnormalities, such as additional valve disease, aorta or other heart conditions – Evaluation of systolic and diastolic function	– Non-invasive imaging – Availability, efficiency, and real-time supply of high temporal and spatial resolution images, without exposure to X-rays	– Hemodynamic parameters, V_peak_, and mean gradient are flow-dependent, which could lead to underestimation of AS at low flow – AVA calculated using the continuity equation may be susceptible to measurement errors, as it depends on three other measurements: left ventricular outflow tract (LVOT) diameter, velocity time integral (VTI) at the LVOT measured by pulsed Doppler, and VTI at the aortic valve – Uncertainty about the true severity of AS due to discordant parameters with lower V_peak_ and gradients indicating less severe AS despite a more severe and restricted AVA
DSE [89,220,221,222]	– Recommended in the case of low-gradient CLF (i.e., stroke volume index < 35 mL/m^2^ and a LVEF < 50%) – Confirmation of true severe AS, and exclusion of pseudo-severe AS by increasing V_peak_ and gradients after dobutamine administration	– Non-invasive imaging – Evaluation of myocardial contractility – Operative risk stratification	– Hemodynamic parameters are flow-dependent – Difficult to assess in patients without flow reserve, who do not increase ejection volume after dobutamine administration and parameters remain unchanged compared to TTE
Exercise Stress Echocardiography [3,4,223,224]	– Provides additional prognostic information by assessing increases in mean pressure gradients and changes in LV function during exercise – Recommended for patients with severe asymptomatic AS to detect symptoms and stratify risk	– Non-invasive imaging – Relatively low cost	– Uninterpretable results with poor ultrasound windows – Some contraindications
CT [31,113,114,225]	– Precise anatomical evaluation – Facilitates pre-procedural planning – Evaluation of prosthetic valve dysfunction and structural valve degeneration	– Availability, reproducibility – Excellent spatial resolution – Low doses of ionizing radiation – Flow-independent – Definition of stenosis severity even in the case of discordant echocardiographic parameters	– Underestimation of AS severity in cases of dominant fibrotic processes
TEE [3,4,226]	– Assess AV morphology and mobility when TTE does not allow for adequate evaluation, when valve repair is being considered, during the periprocedural period, or even pre-TAVR – Precise LVOT measurement	– High spatial resolution	– Invasive imaging modality – Fasting patient – It may be necessary to perform a trans-gastric view – Some contraindications
CMR [3,4,226,227]	– Volume and mass quantification – Evaluation of different chambers – Assessment of AS severity – Quantification of myocardial	– Without radiation – Provides optional information in addition to anatomical data – May serve as a non-invasive alternative to transesophageal echocardiography or catheterization in certain cases of discordant grading on standard evaluation (by TTE and CT)	– Not recommended in guidelines – Costly imaging modality – Long image acquisition and analysis times – Certain contraindications – Limited accessibility

AS = aortic stenosis; AV = aortic valve; AVA = aortic valve area; DSE = dobutamine stress echocardiography; CLF: classical low flow; CMR = Cardiac Magnetic Resonance; CT = computed tomography; LV = left ventricle; LVEF = left ventricle ejection fraction; TAVR = transcatheter aortic valve replacement; TEE = transesophageal echocardiography; TTE = transthoracic echocardiography; V_peak_ = peak aortic jet velocity.

**Table 2:** New emerging imaging modalities for the management of AS.

	Indication/Diagnostic	Advantages	Limits
Contrast-enhanced CT [90,119,228]	– Quantification of both calcified and non-calcified plaques, i.e., fibrous plaques. – Assessment of the myocardium, including measurement of extracellular volume and GLS	– May be preferred over the non-contrast technique, especially in cases where fibrosis is a significant contributor to valve obstruction.	– With contrast – Some contraindications
PET scan [229,230]	– Evaluation of myocardial blood flow, function, and metabolism	– Non-invasive – Improved image quality (especially in obese patients) – High temporal and spatial resolution – Relatively short imaging protocols	– Less available – Costly imaging modality – Some contraindications
CT angiography [231,232]	– Used in the pre-interventional evaluation of TAVR to achieve the following: Determine the access route and size of the aortic annulus. Evaluate coronary anatomy. Quantify calcified and non-calcified coronary plaques. – Select optimal valve bioprosthesis size	– Non-invasive imaging – High temporal resolution – High diagnostic accuracy	– With contrast – Some contraindications

CT = computed tomography; GLS = global longitudinal strain; TAVR = transcatheter aortic valve replacement; PET = positron emission tomography.

**Table 3:** Interventional treatment of AS.

	SAVR [3,4]	TAVR [3,4]	ROSS [3,4,233,234,235,236,237,238,239]
Indications	- Reference procedure for the treatment of AS - Patient aged < 65 years or with life expectancy > 20 years - Patients with severe asymptomatic AS and an abnormal exercise test, very severe AS, rapid progression of AS, or a high BNP level - Stenotic valve is replaced with a bioprosthetic or mechanical valve - Patients in whom anticoagulation is contraindicated should not be candidates for mechanical AVR - Mechanical AVR is recommended for patients aged < 50 years with no contraindication to anticoagulation - Bioprosthesis AVR is recommended for patients > 65 years	- Older patients (age > 80 years or >75 years, AHA(4) and ESC(3) guidelines respectively) with severe AS, or those with life expectancy of less than 10 years - Patients with severe AS and high surgical risk	- Stenosic AVR with the patient’s own pulmonary valve (autograft) and reconstruct the right ventricular ejection pathway using a pulmonary homograft - Young patients with a life expectancy > 15 years, an active or athletic lifestyle and normal aortic dimensions, those at risk of PPM, patients in whom anticoagulant therapy is contraindicated, and women considering pregnancy - Complex procedure - Disadvantages: need for re-intervention of the homograft mainly due to neoartic valve regurgitation, progressive dilatation of the aorta, aortic root or autograft and its association with increased surgical risk - Advantages: excellent valve hemodynamics, no need for anticoagulation and the associated risk of complications or bioprosthesis deterioration, and improved quality of life for patients

AS = aortic stenosis; AVR = aortic valve replacement; BNP = Brain Natriuretic Peptide; SAVR = surgical aortic valve replacement; TAVR = transcatheter aortic valve replacement; PPM = prosthesis–patient mismatch.

## Abbreviations

AS	aortic stenosis
AVC	aortic valve calcification
AVR	aortic valve replacement
BAV	bicuspid aortic valve
CLF	classical low flow
CMR	Cardiac Magnetic Resonance
CT	computed tomography
EOA	effective orifice area
EOAi	indexed effective orifice area
HFpEF	Heart Failure with preserved Ejection Fraction
HFrEF	Heart Failure with reduced Ejection Fraction
LGE	late gadolinium enhancement
LV	left ventricle
LVEF	left ventricular ejection fraction
PET scan	positron emission tomography scan
PLF	paradoxical low flow
SAVR	surgical aortic valve replacement
TAVR	transcatheter aortic valve replacement
TTE	transthoracic echocardiography
TEE	transesophageal echocardiography
V_peak_	peak aortic jet velocity
